# When and how can we predict adaptive responses to climate change?

**DOI:** 10.1093/evlett/qrad038

**Published:** 2023-11-29

**Authors:** Mark C Urban, Janne Swaegers, Robby Stoks, Rhonda R Snook, Sarah P Otto, Daniel W A Noble, Maria Moiron, Maria H Hällfors, Miguel Gómez-Llano, Simone Fior, Julien Cote, Anne Charmantier, Elvire Bestion, David Berger, Julian Baur, Jake M Alexander, Marjo Saastamoinen, Allan H Edelsparre, Celine Teplitsky

**Affiliations:** Department of Ecology and Evolutionary Biology and Center of Biological Risk, University of Connecticut, Storrs, CT, United States; Laboratory of Evolutionary Stress Ecology and Ecotoxicology, University of Leuven, Leuven, Belgium; Laboratory of Evolutionary Stress Ecology and Ecotoxicology, University of Leuven, Leuven, Belgium; Department of Zoology, University of Stockholm, Stockholm, Sweden; Biodiversity Research Centre, Department of Zoology, University of British Columbia, Vancouver, BC, Canada; Division of Ecology and Evolution Research School of Biology, The Australian National University, Canberra, ACT, Australia; Institute of Avian Research, Wilhelmshaven, Germany; Department of Evolutionary Biology, Bielefeld University, Bielefeld, Germany; Nature Solutions Unit, Finnish Environment Institute SYKE, Helsinki, Finland; Department of Environmental and Life Sciences, Karlstad University, Karlstad, Sweden; Institute of Integrative Biology, ETH Zurich, Zurich, Switzerland; Laboratoire Évolution and Diversité Biologique (EDB), UMR5174, CNRS, IRD, Université Toulouse III Paul Sabatier, Toulouse, France; Centre d’Ecologie Fonctionnelle et Evolutive, Université de Montpellier, CNRS, EPHE, IRD, Montpellier, France; Station d’Ecologie Théorique et Expérimentale, CNRS, Moulis, France; Department of Ecology and Genetics, Uppsala University, Uppsala, Sweden; Organismal and Evolutionary Biology Research Programme, Faculty of Biological and Environmental Sciences, University of Helsinki, Helsinki, Finland; Institute of Integrative Biology, ETH Zurich, Zurich, Switzerland; Organismal and Evolutionary Biology Research Programme, Faculty of Biological and Environmental Sciences, University of Helsinki, Helsinki, Finland; Helsinki Institute of Life Science, University of Helsinki, Helsinki, Finland; Department of Ecology and Evolutionary Biology, University of Toronto, Toronto, ON, Canada; Centre d’Ecologie Fonctionnelle et Evolutive, Université de Montpellier, CNRS, EPHE, IRD, Montpellier, France

**Keywords:** global change, climate change, evolvability, prediction, adaptation, evolutionary rescue

## Abstract

Predicting if, when, and how populations can adapt to climate change constitutes one of the greatest challenges in science today. Here, we build from contributions to the special issue on evolutionary adaptation to climate change, a survey of its authors, and recent literature to explore the limits and opportunities for predicting adaptive responses to climate change. We outline what might be predictable now, in the future, and perhaps never even with our best efforts. More accurate predictions are expected for traits characterized by a well-understood mapping between genotypes and phenotypes and traits experiencing strong, direct selection due to climate change. A meta-analysis revealed an overall moderate trait heritability and evolvability in studies performed under future climate conditions but indicated no significant change between current and future climate conditions, suggesting neither more nor less genetic variation for adapting to future climates. Predicting population persistence and evolutionary rescue remains uncertain, especially for the many species without sufficient ecological data. Still, when polled, authors contributing to this special issue were relatively optimistic about our ability to predict future evolutionary responses to climate change. Predictions will improve as we expand efforts to understand diverse organisms, their ecology, and their adaptive potential. Advancements in functional genomic resources, especially their extension to non-model species and the union of evolutionary experiments and “omics,” should also enhance predictions. Although predicting evolutionary responses to climate change remains challenging, even small advances will reduce the substantial uncertainties surrounding future evolutionary responses to climate change.

## Introduction

Climate change is already altering the distribution, abundance, and traits of species and is expected to produce severe future impacts, including extinctions, as the Earth warms further (Cahill et al., 2012; Chen et al., 2011; Maclean & Wilson, 2011; Parmesan, 2006; Urban, 2015). These biodiversity changes could profoundly affect ecosystems and reduce services to humans (IPCC, 2014; Scheffers et al., 2016; Steffen et al., 2015). Preventing the most damaging effects of climate change requires accurate predictions of impacts so that limited conservation resources can be marshaled to design efficient ways to protect biodiversity (Gaitán‐Espitia & Hobday, 2021; Parmesan, 2006; Rockström et al., 2009; Urban et al., 2016).

Adaptive evolution offers an important means for surviving climate change, especially for species with limited dispersal ability or phenotypic plasticity (Gougherty et al., 2021; Merilä & Hendry, 2014; Nadeau & Urban, 2019; Scheffers et al., 2016; Sgrò et al., 2011). Some natural populations have adapted to climate change (Bonnet et al., 2019; Franks et al., 2007; Geerts et al., 2015; Moiron et al., 2023; Singer & Thomas, 1996), whereas others lack the genetic variation needed to adapt fast enough to keep pace with climate change (Fitzpatrick & Keller, 2015; Hoffmann et al., 2003; 2021). Although fundamental for predicting biological impacts from climate change, evolution remains one of the most challenging biological dynamics to predict (Campbell et al., 2017).

Predicting if, when, and how populations can adapt to climate change constitutes one of the greatest challenges in science today. To understand the scope of the problem and its solutions, we build upon insights from this special issue, a poll of its authors, and recent literature to explore the limits and opportunities for predicting adaptive responses to climate change. We review the predictability of five core components of adaptive responses: traits and plasticity, natural selection, genetic variation, evolutionary responses to selection, and population dynamics (Table 1). For each component, we develop a road map for what might be currently predictable, predictable with future research, and what might remain unpredictable despite our best efforts. We then highlight ways to improve future predictions.

**Table 1.** Summary of key questions, what can be predicted now, what is needed to facilitate better predictions, and what we can predict in the future about adaptations to climate change.

	Key question(s)	What can we predict now?	What do we need?	What can we predict in the future?
Traits	What are the most relevant traits involved and what is the role of plasticity in climate change adaptation?	◦ Expression of key physiological and life-history traits ◦ Relationship between temperature and performance ◦ Shifts in phenology and ranges ◦ Responses in dispersal-related traits, drought tolerance	◦ Among population trait values and plasticity estimates combining biotic and abiotic factors ◦ Common garden/transplant experiment on a wider range of traits ◦ Novel and variable environment assessments ◦ Novel techniques (tags, monitors, tracking systems)	◦ How ecological interactions impact climate change adaptation ◦ Trait changes along gradients ◦ Contributions of evolution vs. plasticity
Selection	How to understand the linkages between trait variation and ﬁtness surfaces in response to multiple interactive drivers of climate change that vary in strength of selection?	◦ Single traits under strong selection ◦ Responses to the single environmental driver, mainly temperature ◦ Strong selection from extreme events ◦ Simple, low-diversity systems	◦ Multi-trait and multi-environment selection experiments, analysis, and visualization tools ◦ Common garden/transplant experiments ◦ Selection on indirect effects, e.g., species interactions	◦ Multidimensional selection surfaces ◦ Responses to other drivers than temperature, such as precipitation ◦ Community responses with few interacting species
Genetic variation	Is there enough genetic variance for climate-related traits to keep pace with rapid climate change?	◦ Responses of large vs. small populations ◦ Responses of well-connected vs. poorly connected populations/species ◦ Responses of traits with simple genetic architecture or conserved genetic pathways	◦ Estimates of evolvability for climate-related traits across populations/environments ◦ Genetic covariance matrixes ◦ Genetic bases of climate-related traits ◦ Cost-effective, powerful and easily applied genomic tools for non-model systems	◦ Changes in adaptive potential across environments/populations ◦ Role of cryptic variation and epigenetic variation
Gene flow and demography	Which demographic parameters are critical for understanding adaptative potential of natural (meta)populations to climate change?	◦ Responses of large vs. small populations ◦ Responses of isolated vs. well-connected populations	◦ Estimates of correlated, nonlinear, and indirect impacts from climate change across populations/environments ◦ Estimates of demographic parameters as functions of changing climate ◦ Aggregated databases of existing information, aggregated monitoring and streamlined trait data	◦ Population-specific responses to climatic drivers ◦ Changes in species abundances or gene flow ◦ General models applicable to groups of species, including understudied ones

## Traits and plasticity

### Overview of challenges

**Figure 1.**
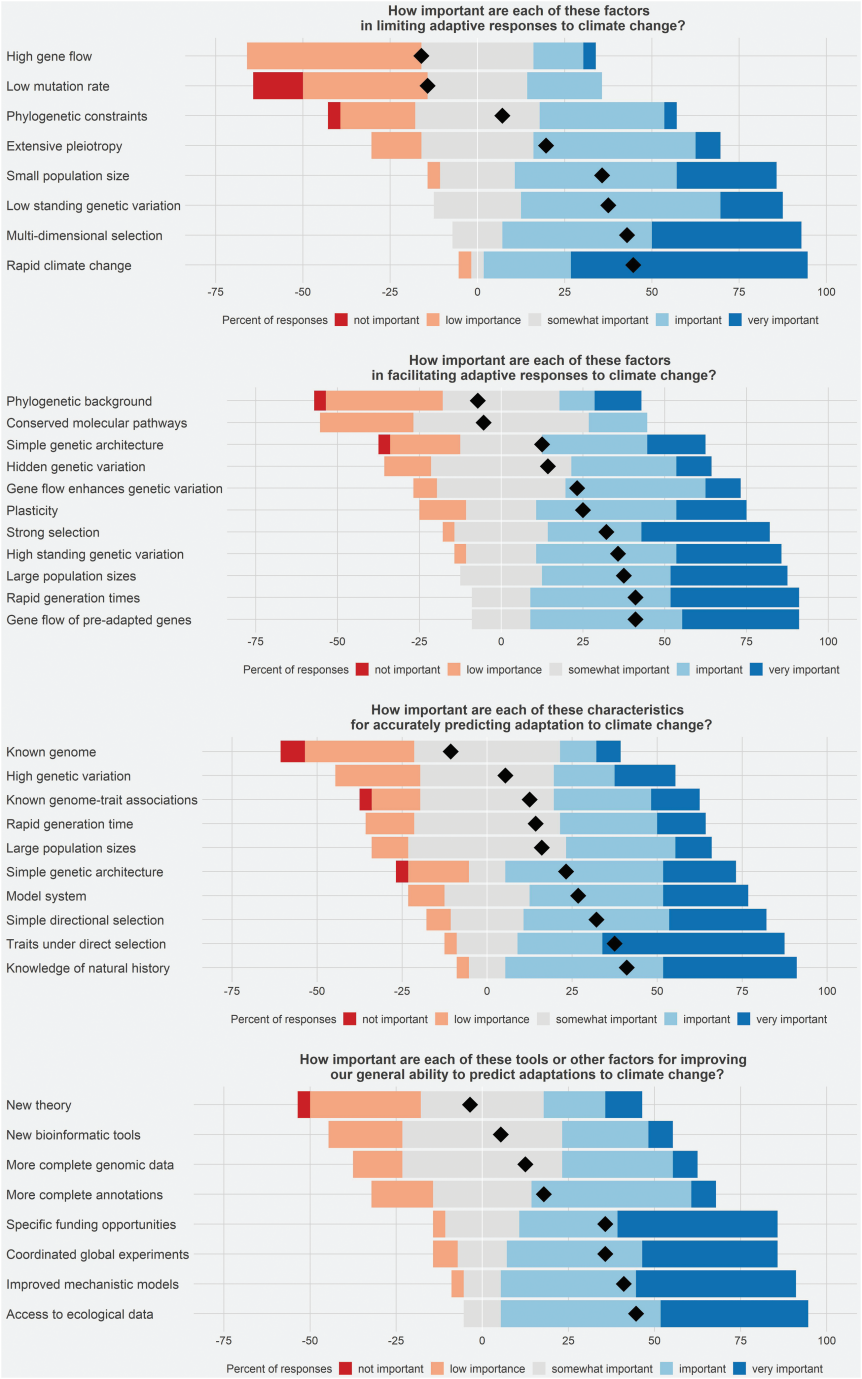
Responses from 28 authors of this special issue on the importance of various limits and opportunities for predicting evolutionary responses to climate change on a scale from 1 (Not important) to 5 (Very important). Responses are indicated by color and medians are indicated by diamonds.

According to our survey of contributors to this special issue, determining which traits to study and their plasticity are among the most important requirements for predicting climate-induced evolution (Figure 1). Climate change affects not only commonly studied traits such as physiology, body size, and life history, but also less-studied traits such as competitive ability. Besides understanding which traits are important, biologists also need to understand when, where, and to what extent plasticity can maintain fitness or evolve. Phenotypic plasticity can be adaptive or maladaptive (Chevin & Hoffmann, 2017), and its effects can depend on both direct and indirect climate effects (Chevin & Lande, 2015; Westneat et al., 2019).

### State of current predictions

Thermal performance (Bennett et al., 2021; Diamond, 2017; Sgrò et al., 2011), drought tolerance (Alberto et al., 2013; Hamann et al., 2018), and traits historically associated with the evolution of expanded range limits such as broader climatic tolerance, faster reproductive rates, and higher dispersal are likely targets of selection from climate change (Dytham, 2009; Lancaster, 2022; Van Bocxlaer et al., 2010). Populations that experienced past climatic variability might retain ancestral phenotypic plasticity (Hällfors et al., 2024, 89–100; Janzen, 1967; Leung et al., 2020; Sunday et al., 2011), but it still might not be sufficient to overcome rapid climate change (Duputié et al., 2015). Adaptive plasticity might sometimes allow populations to persist long enough for adaptations to evolve (Anderson & Song, 2020), but it can also prevent adaptive evolution by buffering selection until it is too late (Robinson & Dukas, 1999; Whitlock, 1996), precluding any general predictions. The relationship between plasticity and genetic variation is also largely unknown. Some highly plastic organisms have limited potential for adaptation (Charmantier et al., 2008; Oostra et al., 2018), while others can adapt readily (Mäkinen et al., 2016; Schaum & Collins, 2014).

### Enabling future predictions

Predicting whether and when plasticity facilitates or impedes adaptive evolution requires identifying the relevant organismal traits, their plasticity, and the relationship between genetic variation and trait plasticity (Duputié et al.; Donelson et al., 2018; Kingsolver & Buckley, 2017; Levis & Pfennig, 2016; Noble et al., 2019; Sgrò et al., 2016). Traits do not operate in isolation, however, and more emphasis needs to be placed on understanding how critical traits covary, how plasticity affects this covariation, and how these relationships alter climate change responses.

Common garden or transplant experiments that replicate future climates can reveal how plasticity varies within and among populations and across multiple traits (Bestion et al., 2023; Stamp & Hadfield, 2020). Performing these experiments at periodic intervals on natural populations or collecting propagules over time can reveal the relative roles of and links between genetic adaptation and plasticity for traits under selection by climate change (Radersma et al., 2020). Improved electronic tags, monitors, remote sensing, and remote camera and video tracking systems can permit researchers to track more traits, at finer scales, and over longer periods (Cui et al., 2020).

## Natural selection

### Overview of challenges

Understanding the agents and strength of natural selection was deemed highly important for predicting climate change adaptation in our poll (Figure 1). However, predicting the manifold effects of climate change on fitness remains challenging. For one, climate-driven selection includes both direct and indirect effects. For example, climate change not only alters weather but also reshuffles species distributions and consequently alters biotic selection (Alexander et al., 2022). Moreover, selection from multiple climate variables often produces multidimensional, nonlinear fitness surfaces (Phillips & Arnold, 1989), and selection can also interact with selection driven by other global changes (Fournier-Level et al., 2019). Climate variables are usually correlated, which could simplify analyses to fewer dimensions, but climate change can also modify these correlations, thus increasing uncertainty (Evans et al., 2018). Climate change forecasts need to be downscaled to match the spatiotemporal scales experienced by organisms, while also recognizing that even finer-scaled microhabitat variation can further modify these experiences (Lenoir et al., 2017; Nadeau et al., 2017; Ziter et al., 2019). The force and direction of natural selection induced by climate often fluctuate, creating a moving target for evolution and increasing predictive uncertainty (Grant & Grant, 2002; Schou et al., 2022; Siepielski et al., 2017). Sexual and natural selection can interact, but little is known about these interactions in natural populations (Baur et al., 2024, 101–113; Gómez-Llano et al., 2024, 149–160; Pilakouta & Ålund, 2021). Lastly, estimating total lifetime fitness is difficult for most species, often requiring biologists to settle for partial or indirect estimates.

### State of current predictions

Predicting selection from climate change depends on understanding how both abiotic and biotic variables affect organismal fitness. For example, understanding how thermodynamic constraints affect protein stability informs predictions for how rising temperatures intensify selection on genomes and affect genetic load (Berger et al., 2021). Generally, biologists can better predict selection from climate change when it alters fitness directly and selection pressures align in their effects across traits and life stages (Etterson & Shaw, 2001; Fisher & McAdam, 2019; Marrot et al., 2017). Weather extremes often cause stronger selection than gradual changes (Campbell-Staton et al., 2017), especially when weather events cross biological thresholds such as thermal limits (Van de Pol et al., 2017). Also, a meta-analysis suggested that precipitation changes induced stronger selection than temperature (Siepielski et al., 2017), emphasizing the multi-dimensionality of climate impacts.

Climate-induced selection is likely more predictable in species-poor systems with fewer indirect effects. For example, selection from drought on beak shape in less-diverse island assemblages of Darwin’s finches was predictable, even though precipitation fluctuations were not (Grant & Grant, 2002). In diverse systems, strong selection might still be predicted for top consumers, for which fitness is often highly sensitive to climate variation (Urban et al., 2017; Zarnetske et al., 2012). However, the many direct and indirect effects of climate change on food web structure and community composition often combine to generate uncertainties that are difficult to resolve.

### Enabling future predictions

Predicting natural selection requires moving beyond simple characterizations of selection along a single axis like temperature and unraveling its multidimensional effects. One way forward is to modify different climate change effects experimentally and estimate the multidimensional selection across traits. Although often constrained to controlled laboratory experiments, manipulating selection in realistic mesocosms or nature is also needed to facilitate more realistic inferences (Bestion et al., 2023; Nadeau & Urban, 2024, 43–55). Transplant experiments offer a particularly appealing approach for estimating realistic shifts in selection along current and future climate gradients (Nooten & Hughes, 2017), especially when situated to incorporate contrasting axes of environmental change, including changing species interactions. Gaps in understanding total fitness will require renewed efforts to measure natural selection throughout the life cycle as well as building demographic models that can account for antagonistic selection across life stages and their potential tradeoffs. Sensor arrays and new technology enable finer-scaled, longer-term data on environmental change and traits under selection over larger spatial and temporal scales (Shi et al., 2014). Overall, understanding selection from climate change in all its manifestations requires an intimate understanding of natural history, which, not surprisingly, was *the* most important factor for improving future predictions in our poll (Figure 1).

## Genetic variation

### Overview of challenges

The surveyed authors highlighted low genetic variation as another important constraint on climate change adaptation (Figure 1). Even when genetic variation exists, it might not fuel evolution that is rapid enough to keep pace with climate change (Botero et al., 2015; Lynch & Lande, 1993). By genetic variation, we refer to additive genetic variation or the multitrait variance–covariance G-matrix, which predicts the adaptive response from climate-induced selection (Sgrò et al., 2011).

To understand adaptive potential, most researchers measure and report heritability, the ratio of additive genetic variation to phenotypic variation. However, heritability is most appropriately applied to situations where selection acts on trait values relative to the distribution of traits in the population (soft selection; Box 1). With climate change, however, selection is expected to depend more on absolute trait values (hard selection), in which case evolutionary responses are expected to depend on additive genetic variance, not heritability (see Box 1; (Gomulkiewicz & Holt, 1995)). This additive genetic variation is usually standardized by the squared trait mean to obtain the scale-invariant measure, *evolvability* (Hansen et al., 2011; Houle, 1992).

**Box 1. Measuring genetic variability in a changing world.**

The evolvability of a population facing a changing environment depends on its genetic variation, but what is the most useful measure of this variation for predicting evolutionary rescue—a population’s additive genetic variance or its heritability?

The relationship between genetic variation and response to selection is well described by classic multilocus quantitative genetic models (Lynch & Walsh, 1998). Famously, the evolutionary response to selection is written in two ways:

$$\begin{array}{l} R=h^{2}S \end{array}$$ (1)

or

$$\begin{array}{l} \Delta z=V_{A}\beta \end{array}$$ (2)

In the first equation, *S* is the selection differential or the distance between the mean trait among individuals that survive and reproduce relative to all individuals, *h*^*2*^ is the heritability or the additive genetic variance *V*_*A*_ divided by the phenotypic variance *V*_*P*_, and R=Δz is the response to selection.

The two equations are mathematically equivalent, but the second equation measures selection relative to the phenotypic variance ($\beta =S/V_{P}$), where *β* represents the coefficient in a regression between fitness and phenotype. Equations 1 and 2 also differ in whether *h*^*2*^ or *V*_*A*_ influences the response to selection. Which is more relevant to predicting how fast a natural population adapts to climate change?

Animal breeders developed the breeder’s equation (Lush, 1945) to predict the change in trait values when selecting some fraction of the population to survive and reproduce based on trait values. If these selected individuals have a mean trait value *S* above the average for the population, heritability predicts the response to selection. Alternatively, if individuals with the most extreme traits along a desirable trait axis are chosen to breed (truncation selection), the selection differential, *S*, equals the intensity of selection (*i*, depending only on the selected fraction) multiplied by the phenotypic standard deviation [$S=i\surd V_{P}$; (Falconer & Mackay, 1996)]. Thus, the more variable the population, the more selected parents will differ from the population, causing stronger selection (*S*) and a larger response (*R*). The breeder’s equation best describes the response to selection when the trait value of an individual relative to the population determines fitness.

When considering selection induced by climate change, however, it is the absolute trait value of an individual, not its trait value relative to the population, that typically determines fitness, as captured by the selection gradient, *β*. For example, all individuals with thermal tolerance curves that match a warming environment might survive, rather than the fraction of the population with the best tolerance curves. In this case, the additive genetic variance, not heritability, determines evolutionary responses to changing environments (Equation 2; Hansen et al., 2011; Houle, 1992).

For example, consider a Gaussian-shaped fitness surface, with an optimal trait value, *θ*, that has shifted away from the mean trait value, *x* (i.e., fitness is given by $exp\;\;\left(-\frac{{\left(\theta -\bar{x}\right)}^{2}}{2\;\omega^{2}}\right)$, where $\omega^{2}$ measures the width of the fitness distribution, with larger values implying weaker selection). The response to selection becomes:

$$\begin{array}{l} R=\;(\theta -\bar{x})\;\frac{V_{A}}{V_{P}+\omega^{2}} \end{array}$$ (3)

(Bulmer, 1980). Because the phenotypic distribution is rarely wider than the fitness distribution ($V_{P}<<\omega^{2}$), the response to selection becomes largely independent of phenotypic variance $\left(R\approx \;\frac{\left(\theta -\bar{x}\right)}{\omega^{2}}V_{A}\right)$ and becomes proportional to the additive genetic variance instead of heritability (Houle, 1992). Similarly, the chance that a population adapts fast enough to persist in the new environment depends primarily on additive genetic variance (Gomulkiewicz & Holt, 1995).

However, if the fitness distribution shifts and narrows substantially such that only a small proportion of the population has any appreciable fitness ($V_{P}>>\omega^{2}$), Equation 3 approaches $R\approx (\theta -\bar{x})h^{2}$. This selection acts more like a breeder: selecting those individuals with traits $S=\theta -\bar{x}$ above the mean, whereby heritability best predicts the response to selection. However, population persistence is also less likely because most individuals have near-zero fitness.

Genetic variances are typically estimated in the lab, but ubiquitous genotype-by-environment interactions compel measurements across multiple natural environments. Quantitative genetic studies conducted in nature have become more common (Bonnet et al., 2022), but they often measure just a few populations, in a few environments, and are taxonomically biased, reducing the confidence that they apply generally. Even when genetic variation exists, a trait might not evolve quickly if genetic correlations among traits do not align with selection or if indirect genetic effects such as maternal effects oppose responses (Baud et al., 2022; Walsh & Blows, 2009).

Despite its importance, genetic variance is seldom incorporated into predictions about climate change risks. Only one of 131 studies that predicted extinction risks from climate change evaluated genetic variance, and it relied on one heritability estimate measured in the lab for one species (Sinervo et al., 2010; Urban, 2015). Yet, when incorporated, evolutionary potential can highlight unrecognized resilience to climate change. For example, after accounting for evolutionary potential, fruit flies were predicted to lose 33% less of their range under future climate change (Bush et al., 2016).

Besides quantitative genetic methods, emerging genomic methods can elucidate genetic architecture and the potential for gene flow or de novo mutations to promote adaptive responses (Bay et al., 2018). Understanding rates of recombination, mutation, gene loss, and phylogenetic constraints can also improve evolutionary predictions. However, these measures are still difficult and costly to estimate for many non-model species and thus were ranked of lower importance in our survey.

### State of current predictions

Evolutionary rescue from climate change depends on initial population size and maladaptation. Large populations facing weaker selection are likely to persist regardless of evolvability, while extremely small populations facing strong selection are likely to face extinction no matter what happens (Gomulkiewicz & Holt, 1995). Only the cases of moderate population size and selection will require sophisticated evolutionary predictions that require estimating additive genetic variance.

The stressful conditions associated with future climate change could reveal cryptic genetic variation for traits (Fisher, 1930) and thereby enhance adaptive potential. Various studies have highlighted the divergent ways in which environmental change can alter genetic variation (Berger et al., 2021; Charmantier & Garant, 2005), generating predictions of increased, decreased, or no change in genetic variance (Hoffmann & Merilä, 1999).

**Figure 2.**
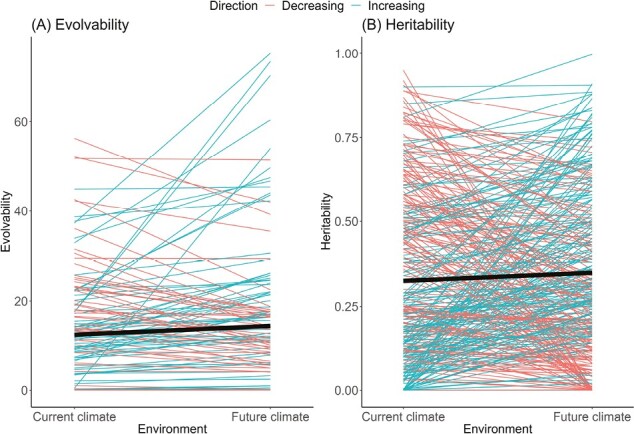
A meta-analysis of 284 estimates of (A) evolvability and (B) 677 estimates of trait heritability measured under current and future environmental changes associated with climate change (heat, drought, acidity, wetness, hypoxia, salinity, carbon dioxide) revealed that individual heritability estimates increased (blue) and decreased (red) in future environments, but overall did not significantly change (black line). We constrained heritabilities to a maximum value of 1 and omitted incomplete data.

To explore how climate change might alter future genetic variation, we conducted a meta-analysis on measurements of additive genetic variation under current and future (stressful) climates (see Supplementary Material for details). We found 10 studies on 15 species that reported 284 evolvabilities and 37 studies on 35 species that reported 677 heritabilities (see Supplementary Table S1 for a list of studies included in the meta-analysis). Although evolvabilities frequently changed between climate treatments in individual studies (Figure 2; mean absolute change = +5.9), the overall mean evolvability did not change, on average, under future climates (Supplementary Tables S2 and S3; +4.1, 95% credible intervals [CIs] = –17.1, 23.8). Heritabilities similarly changed individually across climate treatments (Figure 2; mean absolute change = +0.21), but the mean heritability did not change in future climates (Supplementary Tables S4–S9; +0.02, 95% CIs = –0.01, 0.06). Trait type (life history, morphology, physiology) and climate treatment (acidity, drought, heat, hypoxia, salinity, precipitation) generally did not affect results, except for higher heritabilities of life-history traits during drought (+0.24, 95% CIs = 0.01, 0.49) and lower evolvabilities of morphology during drought (–10.5, 95% CIs = –18.2, –2.7). Another meta-analysis focused on general stress responses and not focused on climate responses (Rowiński & Rogell, 2017) likewise found no overall change in heritability in more stressful environments, but the study found an increased additive genetic variance and phenotypic variance for life-history traits. The difference between these results and ours deserves further investigation to determine if climate stress produces different responses than general stress.

Evolvabilities and heritabilities in our meta-analysis averaged 6.1 (95% CIs = 1.2, 10.6) and 0.32 (0.14, 0.49), respectively, indicating an overall optimistic level of adaptability to future climate change. Although these studies are skewed toward common and tractable species, the results match larger reviews where heritabilities averaged 0.37 (Mousseau & Roff, 1987). Thus, we find reasonable additive genetic variances that could frequently facilitate adaptation to climate change. However, these genetic variances often change unpredictably under future conditions, contributing to high uncertainty.

Species with larger and more connected populations generally should harbor greater genetic variation (Campbell et al., 2017), although empirical results from natural populations are mixed (Wood et al., 2016). Gene flow can facilitate adaptations to climate change by supplying adaptive genes or producing new adaptive combinations (Sexton et al., 2011)—the most important mechanism facilitating adaptive responses according to our poll. Along climate gradients, gene flow from interior populations could facilitate adaptations to changing climate conditions at range edges (Kottler et al., 2021; Lee‐Yaw et al., 2016), as recently demonstrated experimentally (Aguilée et al., 2016; Bontrager & Angert, 2019). In contrast, gene flow from cooler regions could swamp adaptations to warming temperatures along trailing range edges (Nadeau & Urban, 2019).

### Enabling future predictions

Ongoing work is needed to measure additive genetic variances for more species, populations, and traits and connect these estimates to climate gradients. These efforts will provide both population-specific estimates and general insights about changes in evolvability across traits, species, and climate gradients.

Meanwhile, genomic advances could provide alternative estimates for predicting climate change adaptation. For example, genotyping individuals can yield accurate estimates of additive genetic variance in natural populations (Bérénos et al., 2014; Stanton‐Geddes et al., 2013; Yang et al., 2017), reducing the current reliance on pedigrees or complex husbandry experiments and facilitating measurements under natural conditions (Gienapp et al., 2017). Genomic methods also can detect loci of large effect underlying the genetic architecture of adaptation in natural systems (Rodrigues et al., 2022), which could better inform evolutionary predictions. Genomic approaches that link selection to environmental variation (e.g., environmental associations, genome scans) also can dissect past responses to climatic variation and suggest the genetic changes needed for future climates (Jones et al., 2012; Louis et al., 2021; Yeaman et al., 2016). For traits characterized by simple genetic architectures, genome-wide association studies can highlight alleles affecting trait evolution, identify vulnerable populations that lack adaptive alleles, and inform which sources of gene flow might rescue vulnerable populations (Bay et al., 2018; Exposito-Alonso et al., 2018, 2019; Gougherty et al., 2021; Ruegg et al., 2018). Because the spatial distribution of adaptive alleles is likely heterogeneous, some populations will require large shifts in allele frequencies to reduce maladaptation. Relating the adaptive genomic composition of populations to current and future climates can estimate this adaptation lag.

Transcriptomic studies also can highlight loci expressed in different environments and connect underlying genes-to-trait variation, potentially indicating the genes under selection or contributing to plasticity (Oomen & Hutchings, 2022). Target genes could be manipulated through selective breeding, knock-outs, or CRISPR to establish the genes-to-trait mapping with certainty, although some of these manipulative practices remain controversial (Gudmunds et al., 2022). Transcriptomics, however, provides less direct information about evolvability than other approaches and is most usefully applied to better-studied species. As insights accumulate, these studies might eventually inform efforts to predict adaptability and the repeatability of evolutionary trajectories for understudied species, assuming conservatism of shared genetic pathways.

We envision that future efforts will leverage complementary resources from quantitative genetics, genomics, and transcriptomics from experiments and observations to advance reliable estimates of the evolutionary potential of natural populations and predict responses to climate change. However, the large sample sizes needed for accurate results, financial tradeoffs with collecting other critical data or implementing conservation measures, and remaining uncertainties caution against relying solely on genomic tools for the near term.

## Evolutionary responses

### Overview of challenges

The few studies to date that have demonstrated evolutionary rescue during climate change can inform potential genetic pathways and provide general insights (Franks et al., 2007; Gonzalez et al., 2013; Hoffmann & Sgro, 2011; Hoffmann et al., 2021). However, most current insights come from lab-based or model systems and thus might not apply broadly to natural populations. In natural systems, the potential to adapt to climate change is often inferred from observations during short-term weather fluctuations or from existing adaptations to climate across landscapes (Hoffmann & Sgro, 2011; Merilä & Hendry, 2014; Urban et al., 2014). Yet, short-term weather fluctuations might not simulate future climates accurately, and adaptive gene flow across landscapes might not rescue local populations fast enough. So far, predictions about evolutionary responses in the wild have usually been inaccurate (Pujol et al., 2018), highlighting the need to deepen our understanding of evolutionary mechanisms and improve the precision of parameter estimates in natural populations.

### State of current predictions

Species with large population sizes, short generation times, and high additive genetic variation likely will adapt more quickly to climate change (Franks et al., 2007; Geerts et al., 2015). Even longer-lived species can adapt to climatic changes if selection is strong and consistent enough, as observed for Darwin’s finches, red deer, and common terns (Bonnet et al., 2019; Grant & Grant, 2002; Moiron et al., 2024, 8–17). However, other species, like the Soay sheep, responded to climate variation primarily via plastic, rather than genetic, responses despite evolutionary potential (Ozgul et al., 2009). Overall, species that have already adapted to climate variation across their range and that disperse well enough to spread adaptive alleles are likely to adapt more easily to future climates, especially if those conditions were encountered in the past.

Ultimately, our ability to predict evolutionary responses relies on understanding the tension between necessity and chance in evolutionary biology (Gould, 1990). Accumulated evidence from parallel evolution experiments and observations suggests that over shorter periods and in response to strong selection analogous with past selection, evolution often produces similar phenotypes, but not always via the same genetic pathways (Abouheif & Wray, 2002; Colosimo et al., 2005; Conte et al., 2012). Over longer periods and in response to novel selection regimes, evolution is less likely to operate in parallel and more likely to require de novo mutations such that evolutionary trajectories become contingent on the existing genetic architecture (Blount et al., 2008; Lenski, 2017; Whitehead et al., 2017). Therefore, we are more confident about predicting evolutionary responses to climate change over shorter periods in response to analog climates and less confident about making longer-term evolutionary predictions under nonanalog conditions.

### Enabling future predictions

We advocate for initiating and maintaining long-term studies and periodic common garden or transplant experiments that record changes in selection, fitness, traits, and genetics. We also advocate for the collection and preservation of seeds or propagules at regular intervals, such as being done in Project Baseline (Etterson et al., 2016), that would support future resurrection experiments that can detect adaptation over time (Etterson et al., 2016; Franks et al., 2018; Geerts et al., 2015; Orsini et al., 2013). Also, experimental evolution followed by genomic sequencing of ancestors and evolved lineages holds promise for understanding the repeatability of evolved climate change responses under natural conditions (Bailey & Bataillon, 2016). Comparing ancient DNA from specimens in museums and herbaria with current-day genomes can also reveal adaptive genetic differences (Hofreiter et al., 2015; Kreiner et al., 2022; Meineke et al., 2018). In situ climate change experiments in nature offer promising ways to evaluate evolutionary responses under natural conditions, assuming that future conditions can be simulated. Accumulated results would facilitate unified sets of predictions across systems and potentially demonstrate common responses across organisms and ecosystems that can inform understudied systems. Additionally, these experiments will likely reveal when, where, and why some systems are predictable while others remain unpredictable despite our best efforts.

Successfully employing these methods requires coordinated efforts among a global community of researchers committed to unifying analytical and predictive frameworks. Synthetic efforts in other disciplines (e.g., climate change, subatomic physics) have succeeded due to the commitment of extensive resources, the formation of global institutions to organize efforts, and the development of strong cultures of collaboration and sharing (Urban, 2019). Facilitating and adopting these practices would similarly promote predictions for climate change evolution. Limited resources are likely the greatest current impediment, and therefore we need to demonstrate and communicate how better evolutionary predictions can directly improve people’s lives (Carroll et al., 2014).

## Population dynamics

### Overview of challenges

A population must persist to adapt to climate change, and therefore, population dynamics should be included in any serious discussion of adaptive evolution in response to climate change. Models of evolutionary rescue suggest that sufficient genetic variation can support population recovery through adaptation (Carlson et al., 2014; Gomulkiewicz & Holt, 1995; Gonzalez et al., 2013). However, small populations could limit this potential. Besides outright persistence, small populations are also expected to maintain less genetic variation and respond less efficiently to selection, but see (Wood et al., 2016) for contrasting empirical examples.

Predicting population persistence requires understanding population sizes, their underlying demographic processes, and how future climates might affect them. Thus, predicting future population trajectories poses many of the same challenges as predicting evolutionary change: The need to understand correlated, nonlinear, and indirect effects on fitness. Unfortunately, many of the demographic parameters needed to project population dynamics are missing or incomplete for all but the most common species (Urban et al., 2016). Vital rates, such as survival, fecundity, and development rate, are often highly plastic and therefore should be measured as functions of climate rather than static means. When available, vital rates are commonly measured on one population even though local adaptation highlights the need for population- and environment-specific estimates (Hoffmann et al., 2021). Vital rates are also often density- and/or frequency-dependent, which can jointly affect population and evolutionary responses (Engen et al., 2020). Demographic responses could also be nonlinear or involve thresholds that are not easily extrapolated based on past or current responses. Population persistence often depends on immigration and emigration, yet dispersal rates and the dynamic regional context of other populations might often be unknown (Urban et al., 2013).

### State of current predictions

Demographic models for making population predictions are well-developed and often accurate if parameterized with high-quality data and not extrapolated into non-analog climate conditions (Crone et al., 2013; Doak et al., 2021). Meanwhile, newer, more flexible integral projection models have expanded these models’ usefulness by incorporating individual trait variation, plasticity, and genetic variation (Buckley et al., 2010; Enquist et al., 2015; Hanski et al., 2017). However, predicting long-term population dynamics remains challenging due to inaccurate parameters and a lack of information on density dependence, interspecific interactions, and overall evolutionary dynamics.

Initially, large populations or many populations linked by dispersal into metapopulations might be resilient unless they decline substantially (Hanski & Gaggiotti, 2004; Massot et al., 2008; Wright, 1978). High dispersal can allow populations to track their climate niche across elevations or latitudes (Chen et al., 2011). Besides adding genetic variation, dispersal can also promote evolutionary rescue by bolstering declining population abundances (Carlson et al., 2014) although these effects might sometimes be transitory (Lotsander et al., 2021). Small populations with limited dispersal are likely to become isolated and face increasing levels of demographic stochasticity that can limit the potential for persistence and adaptive evolution (Nadeau & Urban, 2019). Additionally, species with low population growth rates and long generation times will be less likely to overcome acute stress from climate change because their numbers cannot rebound fast enough (Pearson et al., 2014). Overall, anything that buffers population declines, including plasticity and dispersal, could provide the time and raw supply of individuals needed to facilitate evolutionary rescue (Gómez-Llano et al., 2024, 149–160).

### Enabling future predictions

With growing evidence for feedback between demography and evolution, eco-evolutionary dynamics models are likely needed to predict joint demographic-evolutionary responses (Pelletier et al., 2007; Walsh & Reznick, 2010). These models can quickly become quite complex and analytically intractable. However, simulations might provide insights until analytical approximations become available. Ultimately, modeling should be thought of as an iterative process that cycles between prediction, validation, and model revision (Dietze et al., 2018).

More detailed and realistic demographic models will require better information on demographic parameters (Urban et al., 2016). Biologists and amateur naturalists increasingly collaborate to record population abundances and traits. GEO-BON is now standardizing and aggregating monitoring data to streamline data collection and make them available for modeling (Pereira et al., 2013). To this end, smaller and more effective transmitters can collect finely resolved demographic data such as survival and dispersal rates. The next step is to evaluate how certain traits, such as physiological stress or body size, might provide early warnings of impending population collapse (Clements et al., 2019; Huey et al., 2012). Aggregating demographic trait data into searchable databases like COMPADRE will facilitate access to and use of these data (Salguero‐Gómez et al., 2016). Combining these existing data with phylogenetic and life-history information can fill gaps for less-studied species (Santini et al., 2016). Concurrently, metapopulation studies are uncovering how multiple populations vary in key demographic traits, and this variation can enable predictions across larger and more relevant spatial scales (Buckley, 2008; Hanski & Saccheri, 2006; Hanski et al., 2017).

## Ten actions to predict evolutionary responses to climate change

Based on our review, we advocate for the following 10 actions to improve the understanding of when and how organisms might adapt genetically to climate change.

1 Expand your knowledge of the natural history of the species and system with which you work. Then develop collaborations with local scientists and community members (Haelewaters et al., 2021) to expand knowledge in understudied systems and strive for a more global representation of species and ecosystems.
2 Design long-term monitoring programs for characterizing population demography, natural selection, and phenotypic and genetic variation through standardized observations, sample collections, and genomic assays across wild populations along climate gradients and as climate changes.
3 Develop experimental assays estimating the relative importance of within- and among-generation plasticity relative to adaptive evolution across varying periods and determining the limits for phenotypic plasticity, when it evolves, and when it interferes with evolution. Link the relative contributions of plasticity versus adaptation in response to climate change to organisms’ ecology and historical exposure to climate variation.
4 Implement and coordinate the systemic deployment of common garden/transplant experiments, resurrection experiments, and evolutionary resurveys across climate gradients to evaluate how climate change alters evolutionary responses and develop the infrastructure to record changes.
5 Estimate gene flow across populations and climate gradients, its contributions to adaptive potential, and its impact on hybrid individuals under future conditions.
6 Contribute data on selection, traits, genetics, evolutionary rates, and population demography to searchable publications and databases to make it available to others. These data, together with phylogenetic information, can later be synthesized to enable generalizations that apply to species and systems with limited information.
7 Perform research aimed at understanding the level of detailed genetic information needed to make accurate predictions about evolutionary responses to climate change. Specifically, answer the question: When do we need measures from quantitative genetics versus genomic sequence data to predict the evolutionary potential of traits under selection?
8 Build and test mechanistic eco-evolutionary models that can incorporate varying levels of genetic detail. These models should be flexible enough to apply to different species, systems, and questions and incorporate levels of genetic specificity from individual loci to quantitative genetics.
9 Understand if and when evolution matters for different traits, populations, species, ecosystems, and questions. The answer is likely context-dependent, but we cannot know the answer until we test models of varying complexity across various systems and assess how well they predict out-of-sample observations. Important questions to answer include: What evolutionary mechanisms are required to make accurate predictions, and when are demographic models without evolution sufficient? Alternatively, is it reasonably accurate to assume a moderate level of evolvability or heritability (e.g., 6 and 0.3, respectively, in our meta-analysis) for all traits in the absence of detailed information?
10 Identify and prioritize (triage) the species and regions that are in greatest need of evolutionary rescue. Decisions should be based on their threat and their importance to ecosystem function (Urban et al., 2017; Zarnetske et al., 2012). Efforts to estimate the potential for evolutionary rescue should be conducted across the phylogenetic spectrum so that we can interpolate insights into closely related species or species with similar traits (Santini et al., 2016). Apply general models and emerging cross-system insights to develop broader management guidelines that promote future resilience, such as conserving or augmenting existing genetic variation, improving connectivity among populations, and increasing population size by maintaining or restoring habitat.

## Conclusions

Despite the many uncertainties associated with predicting evolutionary responses to climate change, the immediate goal is to make better predictions. Therefore, we should not let perfection become the enemy of good. However, we must also be humble. Our predictions will only be as good as data quality and uncertainty dictate. That means estimating all forms of uncertainty, including parameters, model choice, and future climate change, and openly discussing this uncertainty. We will eventually need to test predictions against future data to see when we were right or wrong. Even if wrong, we will have learned much and can use this information to improve future models. As we learn how to make better predictions, we should treat early predictions with caution and simultaneously promote both the specific mitigation actions suggested by models and more universal mitigation actions that broadly maintain natural ecological and evolutionary processes and thereby lend restorative powers during and (hopefully) after climate change.

The potential for evolutionary rescue offers a reason for optimism in the face of changing climates. Although we still know little about when evolution might rescue populations, progress is being made. Sometimes we can predict evolution better than commonly thought given improved data availability and information about which traits and gene complexes might evolve. Based on this growing body of evidence, we conclude that predictions about future evolutionary responses to climate change are becoming more certain, especially for well-studied ecosystems with direct climate change impacts, populations with traits predictably mapped to genetics, and species for which climate change more directly affects fitness.

**Figure 3.**
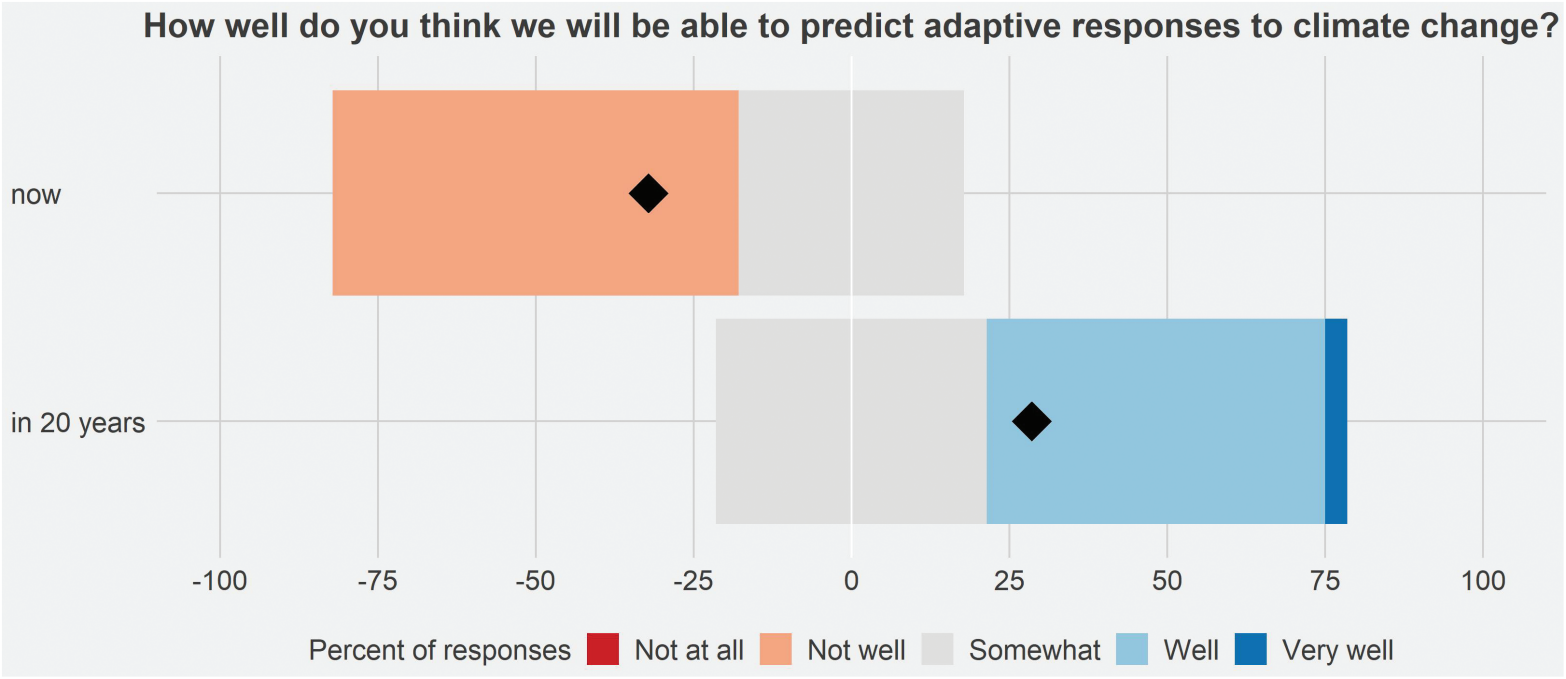
Responses from 28 authors of this special issue to the question, “How well do you think we will be able to predict adaptive responses to climate change right now or as we gain more knowledge in the next 20 years?” on a scale from 1 (Not at all) to 5 (Very well). Responses are indicated by color and medians are indicated by diamonds.

Inspiring hope for the future, many authors in this special issue were optimistic about the ability to predict future adaptive responses to climate change (Figure 3). Overall, authors rated this ability as moderate now (median = 3 of 5, where 5 means very well) and even better (median = 4) in 20 years. Hence, even a group of careful and skeptical evolutionary biologists think that better predictions of evolutionary responses to climate change are possible now and will become even better in the future.

## Supplementary Material

qrad038_suppl_Supplementary_Material

## Data Availability

All data used in this manuscript is available at DOI: 10.5281/zenodo.8233424.
