# Supplementary material for: When and how can we predict adaptive responses to climate change?
Source: Evol Lett. 2023 Nov 29;8(1):172–87. doi: 10.1093/evlett/qrad038 (PMC10872164; doi:10.1093/evlett/qrad038)
Supplement: qrad038_suppl_Supplementary_Material [file qrad038_suppl_supplementary_material.pdf]

Supplementary Materials

for

When and how can we predict adaptive responses to climate change?

Mark C. Urban, Janne Swaegers, Robby Stoks, Rhonda R. Snook, Sarah P. Otto, Daniel W. A. Noble, Maria Moiron, Maria Hällfors, Miguel Gómez-Llano, Simone Fior, Julien Cote, Anne Charmantier, Elvire Bestion, David Berger, Julian Baur, Jake M. Alexander, Marjo Saastamoinen, Allan Edelsparre, Celine Teplitsky

Supplementary methods

Investigating changes in heritability expected under climate change

We aimed to assess whether conditions expected under climate change would affect evolutionary potential. For that purpose, we searched on Web of Science on 26/04/2022 for articles using keywords "heritability", “additive genetic variance”, or “evolvability” associated with either “climate change”, “global warming”, or “extreme climatic event.”

This search led to 535 references, the list was reduced to 97 studies based on reading of the abstract, retaining publications where heritabilities estimates were given under at least two conditions (so this excludes estimates of heritability of tolerance), and including effects expected under climate change such as increased temperature but also changes in precipitation/humidity; or increased CO2 and acidity for aquatic environments. For each study, one reviewer noted heritability, evolvability and additive genetic variance when available, the type of stress (heat, drought, acidity, wetness, hypoxia, salinity, carbon dioxide), the type of heritability (broad vs narrow sense) and the trait type (life history, morphology, physiology, behavior). When reported, 95% confidence intervals were transformed into standard errors using the formula, $\frac{(95\%CI_{High}-95\%CI_{Low})}{2*1.96}$. When compiling the data, we removed studies investigating the heritability of tolerance, providing a single heritability estimate by pooling together data from multiple environments, or not clearly indicating the reference point with future environments. Evolvability was measured as additive genetic variance divided by trait mean (Houle, 1992).

The analyses were run using MCMCglmm (Hadfield, 2010) with a Gaussian distribution. We analyzed the raw values and tested for a difference between normal and future climates. All models included organism class and study number as random effects. Although the model including only the intercept never had the best support, we found no significant effects of stress from climate change on heritability or evolvability estimates.

Table S1: Studies included in the heritability and evolvability meta-analysis.

Study	Species		Class	Order	Factor	Type of h²	error type	Reference
1	fruit fly	*Drosophila melanogaster*	Insecta	Diptera	temperature	broad sense	none	(Rodrigues et al. 2022)
2	Branching coral	*Acropora cervicornis*	Anthozoa	Scleractinia	temperature*acidity	broad sense	95% CI	(Muller et al. 2021)
3	Atlantic salmon	*Salmo salar*	Actinopterygii	Salmoniformes	temperature	narrow sense	95% CI	(Debes et al. 2021)
4	Eastern oyster	*Crassostrea virginica*	Bivalvia	Ostreida	salinity	narrow sense	95% CI	(Griffiths et al. 2021)
5	partridge pea	*Chamaecrista fasciculata*	Eudicots	Fabales	rain	narrow sense	none	(Peschel et al. 2021)
	Thale cress	*Arabidopsis thaliana*	Eudicots	Brassicales	temperature	narrow sense	none	
	Common duckmeat	*Spirodela polyrhiza*	Monocots	Alismatales	temperature	narrow sense	none	
	European beech	*Fagus sylvatica*	Eudicots	Fagales	temperature	narrow sense	none	
	squinting bush brown	*Bicyclus anynana*	Insecta	Lepidoptera	temperature	narrow sense	none	
6	Red flour beetle	*Tribolium castaneum*	Insecta	Coleoptera	temperature	narrow sense	none	(Fischer et al. 2020)
7	fruit fly	*Drosophila melanogaster*	Insecta	Diptera	temperature	narrow sense	se	(Zwoinska et al. 2020)
8	fungus	*Neurospora crassa*	Sordariomycetes	Sordariales	temperature	broad sense	95% CI	(Moghadam et al. 2020)
9	woodland strawberry	*Fragaria vesca*	Eudicots	Rosales	drought	broad sense	se	(De Kort et al. 2020)
10	Annual phlox	*Phlox drummondii*	Eudicots	Ericales	drought	broad sense	se	(Suni et al. 2020)
11	Drummond's rockcress	*Boechera stricta*	Eudicots	Brassicales	drought*nutrient	broad sense	95% CI	(Mac Tavish and Anderson 2020)
12	eastern cottonwood	*Populus deltoides*	Eudicots	Malpighiales	flooding	broad sense	se	(Rodríguez et al. 2020)
13	Frémont's goldfields	*Lasthenia fremontii*	Eudicots	Asterales	ECE / flooding regime	narrow sense	none	(Torres-Martínez et al. 2019)
14	Encino	*Quercus oleoides*	Eudicots	Fagales	watering regime	narrow sense	se	(Ramírez-Valiente et al. 2019)
15	California Grunion	*Leuresthes tenuis*	Actinopterygii	Atheriniformes	ocean acidification	narrow sense	95% CI	(Tasoff and Johnson 2018)
16	lesser valley coral	*Platygyra daedalea*	Hexacoralia	Scleractinia	temperature	narrow sense	none	(Kirk et al. 2018)

17	Quacking frog	*Crinia georgiana*	Amphibia	Anura	water depth	narrow sense	none	(Rudin-Bitterli et al. 2018)
18	intertidal tubeworm	*Galeolaria caespitosa*	Polycaeta	Sabellida	temperature	narrow sense	none	(Chirgwin et al. 2018)
19	brown anole lizards	*Anolis sagrei*	Reptilia	Squamata	temperature	narrow sense	se	(Logan et al. 2018)
20	intertidal tubeworm	*Galeolaria caespitosa*	Polycaeta	Sabellida	temperature	narrow sense	none	(Chirgwin et al. 2017)
21	spiny chromis	*Acanthochromis polyacanthus*	Actinopterygii	Pomacentridae	temperature	narrow sense	se	(Munday et al. 2017)
22	Black alder	*Alnus glutinosa*	Eudicots	Fagales	temperature	broad sense	se	(De Kort et al. 2016)
23	fruit fly	*Drosophila birchii*	Insecta	Diptera	temperature	narrow sense	se	(van Heerwaarden et al. 2016)
24	intertidal tubeworm	*Galeolaria caespitosa*	Polycaeta	Sabellida	temperature	narrow sense	none	(Chirgwin et al. 2015)
25	fruit fly	*Drosophila melanogaster*	Insecta	Diptera	temperature	narrow sense	95% CI	(Kristensen et al. 2015)
26	Three-spined Stickleback	*Gasterosteus aculeatus*	Actinopterygii	Gasterosteiformes	temperature	narrow sense	95% CI	(Shama et al. 2014)
29	sea urchin	*Strongylocentrotus purpuratus*	Echinoidae	Echinoida	ocean acidification	broad sense	95% CI	(Kelly et al. 2013)
30	fruit fly	*Drosophila simulans*	Insecta	Diptera	temperature	narrow sense	SE	(van Heerwaarden and Sgrò 2013)
31	Atlantic salmon	*Salmo salar*	Actinopterygii	Salmoniformes	hypoxia	narrow sense	95% CI	(Côte et al. 2012)
32	Crawling frog	*Pseudophryne guentheri*	Amphibia	Anura	drought	narrow sense	none	(Eads et al. 2012)
33	Patagonian cypress	*Austrocedrus chilensis*	Pinopsida	Pinales	drought	broad sense	se	(Aparicio et al. 2012)
34	fruit fly	*Drosophila melanogaster*	Insecta	Diptera	temperature	narrow sense	95% CI	(Ketola et al. 2012)
35	damselfly	*Ischnura elegans*	Insecta	Odonata	temperature	narrow sense	95% CI	(Shama et al. 2011)
36	great tit	*Parus major*	Aves	Passeriformes	temperature	narrow sense	none	(Husby et al. 2010)
37	Arctic charr	*Salvelinus alpinus*	Actinopterygii	Salmoniformes	Temperature	broad sense	none	(Janhunen et al. 2010)
39	great tit	*Parus major*	Aves	Passeriformes	Temperature	narrow sense	se	(Garant et al. 2008)
41	Partridge pea	*Chamaecrista fasciculata*	Eudicots	Fabales	drought	narrow sense	none	(Etterson 2004)
42	Scots pine	*Pinus sylvestris*	Pinopsida	Pinales	Temperature & water limitation	broad sense	se	(Sonesson and Eriksson 2000)

Evolvability meta-analysis results

Table S2: Model selection for evolvability analysis

	Intercept	Stress	Type of trait	Type of stress	Type of h²	Type of experiment	Stress*Type of stress	Stress*Type of trait	DIC
M10	x		x					x	2154.7
M9	x		x						2166.5
M0	x								2196.8
M3	x				x				2197.4
M1	x	x							2197.8
M6	x	x			x				2197.9
M4	x					x			2198.7
M7	x	x				x			2199.4
M2	x			x					2201.6
M5	x	x		x					2202.1
M8	x	x		x			x		2208.3

Note - The analysis was run on a data set comprising 284 observations for 10 studies across 15 species.

Table S3: Summary of best model (M10) for evolvability analysis

	post.mean	95%CI - Low	95%CI - High	eff.samp	CI
Class	0.000	0.000	0.000	235.8	
Study	215.600	34.460	512.900	53.24	
units	109.600	90.680	127.900	1000	
	post.mean	95%CI - Low	95%CI - High	eff.samp	pMCMC
(Intercept)	4.123	-17.106	23.766	81.91	0.724
TraitTypeLHT	0.585	-7.047	8.210	802.56	0.886
TraitTypeMorphology	0.017	-7.308	6.545	1000	0.974
TypeStressdrought	7.159	-22.052	30.779	62.39	0.59
TypeStressheat	1.007	-16.987	18.855	128.1	0.922
TypeStresswet	12.890	-14.379	41.224	59.28	0.37
TraitTypeLHT:TypeStressdrought	8.301	-2.054	17.459	619.59	0.104
**TraitTypeMorphology:TypeStress: drought**	**-10.522**	**-18.245**	**-2.683**	**1000**	**0.01**

Note - Morphological traits have lower evolvability in drought experiments, but not in relation to stress itself.

Heritability meta-analysis results

Table S4: Model selection for heritability analysis including all estimates.

	Intercept	Stress	Type of trait	Type of stress	Type of h^2	Type of experiment	Stress*Type of stress	Stress*Type of trait	DIC
M10	x	X	x					x	41.8
M2	x			x					50.6
M5	x	x		x					50.6
M8	x	x		x			x		57.0
M0	x								60.5
M9	x		x						60.5
M1	x	x							61.1
M3	x				x				61.2
M6	x	x			x				61.3
M4	x					x			62.5
M7	x	x				x			63.2

Note - Many studies did not report errors around heritability estimates, so we first looked at the effect of stress and other factors on heritability. This analysis relied on 677 estimates from 37 studies across 35 species. The best model is the one with lowest DIC.

Table S5: Output from the best heritability model (M10). Life history traits had larger heritability under drought (aridity experiments in plants + pond drying in amphibians).

Random effects	post.mean	95%CI - Low	95%CI - High	eff.samp	
Class	0.03872	0.002975	0.08364	1000	
Study	0.02291	0.006543	0.04442	908.7	
Units	0.05871	0.05216	0.06509	913.1	
**Fixedeffects**	**post.mean**	**95%CI - Low**	**95%CI - High**	**eff.samp**	**pMCMC**
(Intercept)	0.27	-0.38	1.01	1000	0.432
TraitTypeLHT	0.15	-0.48	0.75	1000	0.616
TraitTypeMorphology	0.29	-0.29	0.93	912.1	0.36
TraitTypePhysiology	0.27	-0.37	0.78	895.9	0.342
TypeStressacidity	-0.24	-0.66	0.20	1000	0.282
TypeStressdrought	-0.21	-0.70	0.23	1000	0.352
TypeStressheat	-0.25	-0.67	0.15	1000	0.228
TypeStresshypoxia	-0.07	-0.67	0.47	1000	0.808
TypeStresslowSalinity	0.10	-0.73	0.176	1111.8	0.77
TypeStresswet	-0.35	-0.89	0.22	1000	0.184
TraitTypeLHT:TypeStressacidity	0.09	-0.30	0.43	1193.9	0.608
TraitTypeMorphology:TypeStressacidity	0.00	-0.30	0.32	1000	0.982
**TraitTypeLHT:TypeStressdrought**	**0.24**	**0.01**	**0.49**	**1000**	**0.042**

TraitTypeMorphology:TypeStressdrought	0.03	-0.23	0.24	1000	0.838
TraitTypeLHT:TypeStressheat	0.18	-0.06	0.46	1000	0.198
TraitTypeMorphology:TypeStressheat	-0.01	-0.27	0.23	1243.1	0.914
TraitTypeLHT:TypeStresshypoxia	-0.16	-0.38	0.08	1000	0.2

Table S6: Model selection for heritability analysis including only studies where standard errors were reported.

	Intercept	Stress	Type of trait	Type of stress	Type of h^2	Type of experiment	Stress*Type of stress	Stress*Type of trait	DIC
M2	x			x					-1703.4
M10	x		x					x	-1698.3
M4	x					x			-1680.7
M5	x	x		x					-1674.1
M3	x				x				-1671.2
M0	x								-1664.3
M1	x	x							-1647.8
M6	x	x			x				-1643.6
M7	x	x				x			-1643.2
M8	x	x		x			x		-1629.3
M9	x		x						-1624.9

Note - Including only studies where standard errors are reported reduced the data set to 536 estimates from 26 studies across 23 species. In this model, we used the mev function in MCMCglmm to account for sampling variance around the estimates. We used a non-informative prior with V = 1 and nu = 0.02.

Table S7: Summary of the best model (M2) for heritability analysis including only studies where standard errors were reported.

	post.mean	95%CI - Low	95%CI - High	eff.samp	CI
Class	0.03561	0.001709	0.1139	1000	
Study	0.08176	0.02869	0.1445	1000	
Units	0.001126	0.000487	0.002017	1000	
	post.mean	95%CI - Low	95%CI - High	eff.samp	pMCMC
(Intercept)	0.5378	-0.1527	1.208	1000	0.134
TypeStressacidity	-0.3074	-0.9546	0.379	1000	0.372
TypeStressdrought	-0.1945	-0.9807	0.5074	1248	0.576
TypeStressheat	-0.2258	-0.8743	0.4295	1000	0.502
TypeStresshypoxia	-0.532	-1.4277	0.3048	1017	0.226
TypeStresslow salinity	0.1141	-0.917	1.1899	1000	0.846
TypeStresswet	-0.1081	-0.9268	0.7351	1000	0.806

Table S8: Model selection for heritability analysis including only studies where standard errors were reported AND restricting to the most common factors: heat and drought.

	Intercept	Stress	Type of trait	Type of stress	Type of h^2	Type of experiment	Stress* Type of stress	Stress* Type of trait	DIC
M4	x					x			-1022.4
M3	x				x				-1020.5
M2	x			x					-1020.0
M0	x								-1018.4
M6	x	x			x				-1001.8
M7	x	x				x			-996.8
M1	x	x							-993.6
M5	x	x		x					-987.1
M10	x		x					x	-982.9
M9	x		x						-973.6
M8	x	x		x			x		-955.6

Note - This analysis was based on 429 observations for 20 studies across 18 species.

Table S9: Summary of best model (M4) for heritability analysis including only studies where standard errors were reported AND restricting to the most common factors: heat and drought.

	post.mean	95%CI - Low	95%CI - High	eff.samp	
Class	0.041	0.002	0.140	1000	
Study	0.075	0.024	0.143	911.4	
Units	0.003	0.001	0.005	1000	
	post.mean	95%CI - Low	95%CI - High	eff.samp	pMCMC
(Intercept)	0.022	-0.601	0.657	1000	0.902
Lab Experiment	0.320	-0.315	0.962	887.4	0.274
Natural variation	0.223	-0.713	1.165	1000	0.622

References

Aparicio, A., Zuki, S., Pastorino, M., Martinez-Meier, A. & Gallo, L. (2012) Heritable variation in the survival of seedlings from Patagonian cypress marginal xeric populations coping with drought and extreme cold. Tree Genetics and Genomes, 8, 801–810.

Chirgwin, E., Marshall, D.J., Sgrò, C.M. & Monro, K. (2017) The other 96%: Can neglected sources of fitness variation offer new insights into adaptation to global change? Evolutionary Applications, 10, 267–275.

Chirgwin, E., Marshall, D.J., Sgrò, C.M. & Monro, K. (2018) How does parental environment influence the potential for adaptation to global change? Proceedings of the Royal Society B: Biological Sciences, 285, 20181374.

Chirgwin, E., Monro, K., Sgrò, C.M. & Marshall, D.J. (2015) Revealing hidden evolutionary capacity to cope with global change. Global change biology, 21, 3356–3366.

Côte, J., Roussel, J.M., Cam, S. Le, Bal, G. & Evanno, G. (2012) Population differences in response to hypoxic stress in Atlantic salmon. Journal of evolutionary biology, 25, 2596–2606.

Debes, P. V., Solberg, M.F., Matre, I.H., Dyrhovden, L. & Glover, K.A. (2021) Genetic variation for upper thermal tolerance diminishes within and between populations with increasing acclimation temperature in Atlantic salmon. Heredity 2021 127:5, 127, 455–466.

Eads, A.R., Mitchell, N.J. & Evans, J.P. (2012) Patterns of genetic variation in desiccation tolerance in embryos of the terrestrial-breeding frog, Pseudophryne guentheri. Evolution, 66, 2865–2877.

Etterson, J.R. (2004) Evolutionary potential of Chamaecrista fasciculata in relation to climate change. I. Clinal patterns of selection along an environmental gradient in the great plains. Evolution; international journal of organic evolution, 58, 1446–1458.

Fischer, K., Kreyling, J., Beaulieu, M., Beil, I., Bog, M., Bonte, D., et al. (2020) Species-specific effects of thermal stress on the expression of genetic variation across a diverse group of plant and animal taxa under experimental conditions. Heredity 2020 126:1, 126, 23–37.

Garant, D., Hadfield, J.D., Kruuk, L.E.B. & Sheldon, B.C. (2008) Stability of genetic variance and covariance for reproductive characters in the face of climate change in a wild bird population. Molecular Ecology, 17, 179–188.

Griffiths, J.S., Johnson, K.M., Sirovy, K.A., Yeats, M.S., Pan, F.T.C., Peyre, J.F. La, et al. (2021) Transgenerational plasticity and the capacity to adapt to low salinity in the eastern oyster, Crassostrea virginica. Proceedings of the Royal Society B: Biological Sciences, 288.

Hadfield, J.D. (2010). MCMC methods for multi-response generalized linear mixed models: the MCMCglmm R package. Journal of Statistical Software 33:1-22.

Heerwaarden, B. van, Malmberg, M. & Sgrò, C.M. (2016) Increases in the evolutionary potential of upper thermal limits under warmer temperatures in two rainforest Drosophila species. Evolution, 70, 456–464.

Heerwaarden, B. van & Sgrò, C.M. (2013) Multivariate analysis of adaptive capacity for upper thermal limits in Drosophila simulans. Journal of evolutionary biology, 26, 800–809.

Houle, D. (1992). Comparing evolvability and variability of quantitative traits. Genetics 130:195-204.

Husby, A., Nussey, D.H., Visser, M.E., Wilson, A.J., Sheldon, B.C. & Kruuk, L.E.B. (2010) Contrasting patterns of phenotypic plasticity in reproductive traits in two great tit (<i>Parus major<i>) populations. Evolution, 64, 2221–2237.

Janhunen, M., Piironen, J. & Peuhkuri, N. (2010) Parental effects on embryonic viability and growth in Arctic charr Salvelinus alpinus at two incubation temperatures. Journal of fish biology, 76, 2558–2570.

Kelly, M.W., Padilla-Gamiño, J.L. & Hofmann, G.E. (2013) Natural variation and the capacity to adapt to ocean acidification in the keystone sea urchin Strongylocentrotus purpuratus. Global Change Biology, 19, 2536–2546.

Ketola, T., Kellermann, V., Kristensen, T.N. & Loeschcke, V. (2012) Constant, cycling, hot and cold thermal environments: strong effects on mean viability but not on genetic estimates. Journal of Evolutionary Biology, 25, 1209–1215.

Kirk, N.L., Howells, E.J., Abrego, D., Burt, J.A. & Meyer, E. (2018) Genomic and transcriptomic signals of thermal tolerance in heat-tolerant corals (Platygyra daedalea) of the Arabian/Persian Gulf. Molecular Ecology, 27, 5180–5194.

Kort, H. De, Mijnsbrugge, K. Vander, Vandepitte, K., Mergeay, J., Ovaskainen, O. & Honnay, O. (2016) Evolution, plasticity and evolving plasticity of phenology in the tree species Alnus glutinosa. Journal of Evolutionary Biology, 29, 253–264.

Kort, H. De, Panis, B., Helsen, K., Douzet, R., Janssens, S.B. & Honnay, O. (2020) Pre-adaptation to climate change through topography-driven phenotypic plasticity. Journal of Ecology, 108, 1465–1474.

Kristensen, T.N., Overgaard, J., Lassen, J., Hoffmann, A.A. & Sgrò, C. (2015) Low evolutionary potential for egg-to-adult viability in Drosophila melanogaster at high temperatures. Evolution, 69, 803–814.

Logan, M.L., Curlis, J.D., Gilbert, A.L., Miles, D.B., Chung, A.K., McGlothlin, J.W., et al. (2018) Thermal physiology and thermoregulatory behaviour exhibit low heritability despite genetic divergence between lizard populations. Proceedings of the Royal Society B: Biological Sciences, 285, 20180697.

Moghadam, N.N., Sidhu, K., Summanen, P.A.M., Ketola, T. & Kronholm, I. (2020) Quantitative genetics of temperature performance curves of Neurospora crassa. Evolution, 74, 1772–1787.

Muller, E.M., Dungan, A.M., Million, W.C., Eaton, K.R., Petrik, C., Bartels, E., et al. (2021) Heritable variation and lack of tradeoffs suggest adaptive capacity in Acropora cervicornis despite negative synergism under climate change scenarios. Proceedings of the Royal Society B, 288.

Munday, P.L., Donelson, J.M. & Domingos, J.A. (2017) Potential for adaptation to climate change in a coral reef fish. Global Change Biology, 23, 307–317.

Peschel, A.R., Boehm, E.L. & Shaw, R.G. (2021) Estimating the capacity of Chamaecrista fasciculata for adaptation to change in precipitation. Evolution, 75, 73–85.

Ramírez-Valiente, J.A., Etterson, J.R., Deacon, N.J. & Cavender-Bares, J. (2019) Evolutionary potential varies across populations and traits in the neotropical oak Quercus oleoides. Tree Physiology, 39, 427–439.

Rodrigues, L.R., McDermott, H.A., Villanueva, I., Djukarić, J., Ruf, L.C., Amcoff, M., et al. (2022) Fluctuating heat stress during development exposes reproductive costs and putative benefits. Journal of Animal Ecology, 91, 391–403.

Rodríguez, M.E., Lauff, D., Cortizo, S. & Luquez, V.M.C. (2020) Variability in flooding tolerance, growth and leaf traits in a Populus deltoides intraspecific progeny. Tree physiology, 40, 19–29.

Rudin-Bitterli, T.S., Mitchell, N.J. & Evans, J.P. (2018) Environmental Stress Increases the Magnitude of Nonadditive Genetic Variation in Offspring Fitness in the Frog Crinia georgiana. https://doi.org/10.1086/699231, 192, 461–478.

Shama, L.N.S., Campero-Paz, M., Wegner, K.M., Block, M. De & Stoks, R. (2011) Latitudinal and voltinism compensation shape thermal reaction norms for growth rate. Molecular Ecology, 20, 2929–2941.

Shama, L.N.S., Strobel, A., Mark, F.C. & Wegner, K.M. (2014) Transgenerational plasticity in marine sticklebacks: Maternal effects mediate impacts of a warming ocean. Functional Ecology, 28, 1482–1493.

Sonesson, J. & Eriksson, G. (2000) Genotypic Stability and Genetic Parameters for Growth and Biomass Traits in a Water × Temperature Factorial Experiment with Pinus sylvestris L. Seedlings. Forest Science, 46, 487–495.

Suni, S.S., Ainsworth, B. & Hopkins, R. (2020) Local adaptation mediates floral responses to water limitation in an annual wildflower. American Journal of Botany, 107, 209–218.

Tasoff, A.J. & Johnson, D.W. (2018) Can larvae of a marine fish adapt to ocean acidification? Evaluating the evolutionary potential of California Grunion (Leuresthes tenuis). Evolutionary applications, 12, 560–571.

Tavish, R. Mac & Anderson, J.T. (2020) Resource availability alters fitness trade-offs: implications for evolution in stressful environments. American Journal of Botany, 107, 308–318.

Torres-Martínez, L., McCarten, N. & Emery, N.C. (2019) The adaptive potential of plant populations in response to extreme climate events. Ecology Letters.

Zwoinska, M.K., Rodrigues, L.R., Slate, J. & Snook, R.R. (2020) Phenotypic Responses to and Genetic Architecture of Sterility Following Exposure to Sub-Lethal Temperature During Development. Frontiers in Genetics, 11, 573.
